# Fuzzy clustering-based feature extraction method for mental task classification

**DOI:** 10.1007/s40708-016-0056-0

**Published:** 2016-09-03

**Authors:** Akshansh Gupta, Dhirendra Kumar

**Affiliations:** 1 School of Computational and Integrative Sciences, Jawaharlal Nehru University, New Delhi, 110067 India; 2 School of Computer and Systems Sciences, Jawaharlal Nehru University, New Delhi, 110067 India

**Keywords:** Brain computer interface, Mental tasks classification, Feature extraction, Empirical wavelet transform, Fuzzy C-means clustering, Feature selection

## Abstract

A brain computer interface (BCI) is a communication system by which a person can send messages or requests for basic necessities without using peripheral nerves and muscles. Response to mental task-based BCI is one of the privileged areas of investigation. Electroencephalography (EEG) signals are used to represent the brain activities in the BCI domain. For any mental task classification model, the performance of the learning model depends on the extraction of features from EEG signal. In literature, wavelet transform and empirical mode decomposition are two popular feature extraction methods used to analyze a signal having non-linear and non-stationary property. By adopting the virtue of both techniques, a theoretical adaptive filter-based method to decompose non-linear and non-stationary signal has been proposed known as empirical wavelet transform (EWT) in recent past. EWT does not work well for the signals having overlapped in frequency and time domain and failed to provide good features for further classification. In this work, Fuzzy c-means algorithm is utilized along with EWT to handle this problem. It has been observed from the experimental results that EWT along with fuzzy clustering outperforms in comparison to EWT for the EEG-based response to mental task problem. Further, in case of mental task classification, the ratio of samples to features is very small. To handle the problem of small ratio of samples to features, in this paper, we have also utilized three well-known multivariate feature selection methods viz. Bhattacharyya distance (BD), ratio of scatter matrices (SR), and linear regression (LR). The results of experiment demonstrate that the performance of mental task classification has improved considerably by aforesaid methods. Ranking method and Friedman’s statistical test are also performed to rank and compare different combinations of feature extraction methods and feature selection methods which endorse the efficacy of the proposed approach.

## Introduction

Brain computer interface (BCI) is a communication system by which a person can send messages or request for basic necessities via his or her brain signals without using peripheral nerves and muscles [1]. It is one of the areas which has contributed to the development of neuron-based techniques to provide solutions for disease prediction, communication, and control [2–4]. Three acquisition modalities have been discussed in the literature [5, 6], viz, invasive (microelectrode array), semi-invasive [electrocorticography (ECoG)], and non-invasive (EEG) for capturing signals corresponding to brain activities. EEG is a widely preferred technique to capture brain activity for BCI system [7, 4] as its ability to record brain signals in a non-surgical manner leading to low cost. *Response to mental tasks* is one of the BCI systems [8], which is found to be more pragmatic for locomotive patients. This system is based on the assumption that different mental activities lead to typical, distinguishable and task-specific patterns of EEG signal. The success of this BCI system depends on the classification accuracy of brain signals. Extraction of relevant and distinct features from EEG signal associated with different mental tasks is necessary to develop an efficient classification model.

In the literature, a number of analytic approaches have been employed by the BCI community for better representation of EEG signal such as band power [9], amplitude values of EEG signals [10], power spectral density (PSD) [11–13], autoregressive (AR), and adaptive autoregressive (AAR) parameters [14]. However, the primary issue with AR modeling is that the accuracy of the spectral estimate is highly dependent on the selected model order. An insufficient model order tends to blur the spectrum, whereas an overly large order may create artificial peaks in the spectrum. In fact, the frequency spectrum of the EEG signal is observed to vary over time, indicating that the EEG signal is a non-stationary signal. As a consequence, such a feature extraction method should be chosen which can model the non-stationary effect in the signal for better representation.

The wavelet transform (WT) [15, 16] is an effective technique that can be used to analyze both time and frequency contents of the signal. However, WT uses some fixed basis mother wavelets, independent of the processed signal, which makes it non-adaptive. Another successful method for feature extraction, empirical mode decomposition (EMD) [17], represents the non-linear and non-stationary signal in terms of modes that correspond to the underlying signal. EMD is a data-driven approach that does not use a fixed set of basis functions, but is self-adaptive according to the processed signal. It decomposes a signal into finite, well-defined, low-frequency and high-frequency components known as intrinsic mode functions (IMFs) or modes.

Due to multi-channel nature of EEG data, the dimensionality of extracted features is very large but the available number of samples per class is usually small in such application. Hence, it suffers from curse-of-dimensionality problem [18], which also leads peaking phenomena in the phase of designing classifier [19]. To overcome this problem, dimensionality reduction using feature selection is suggested in the literature [20].

In this paper, a two-phase approach has been used to determine a reduced set of relevant and non-redundant features to solve the above-mentioned issues. In the first phase, features in terms of eight different parameters are extracted from the decomposed EEG signal using empirical wavelet transform (EWT) or the proposed FEWT. In the second phase, the multivariate filter feature selection approach is employed to select a set of relevant and non-redundant features. To investigate the performance of different combinations of the two feature extraction and multivariate feature selection methods, experiments are performed on a publicly available EEG data [4].

The rest of the paper is organized as follows: The EWT have been discussed briefly in Sect. 2. The proposed feature extraction technique for mental task classification and Fuzzy c-means (FCM) algorithm have been discussed in Sect. 3. Multivariate feature selection methods are included in Sect. 4. Description of experimental setup and results are discussed in Sect. 5. Finally, Sect. 6 includes conclusions and future work.

## Empirical wavelet transform

The nature of the EEG is non-linear and non-stationary [21]. To deal this nature of the EEG signal, in recent past, a fixed basis function based on the WT [22, 23] and an adaptive filter-based EMD methods have been applied [24, 25]. The major concern of EMD method is the lack of mathematical theory [26]. Combining properties of these two methods, recently Gilles [26] has proposed a new adaptive basis transform called EWT to extract the mode of amplitude-modulated–frequency-modulated (AM-FM) signal. The method to build a family of adaptive (empirical) wavelets of the signal to be processed is the same as the formation of a set of bandpass filters in Fourier spectrum. The idea to achieve the adaptability is the dependency of filter’s supports on the location of the information in the spectrum of the signal [26].

Let $\omega$ denote the frequency, which belongs to a segmented of *N* continuous segment, Fourier support, $\left[ o,\pi \right]$. Further $\omega _{n}$ denotes the limit between each segment ($\omega _{0}=0$ and $\omega _{N}=\pi)$ and $\Lambda _{n}=\left[ \omega _{n-1},\omega _{n}\right]$ denotes a segment such that $\bigcup _{n=1}^{N}\Lambda _{n}=\left[ 0,\pi \right]$. It is assumed that the each segment having a transition phase, which is centered around $\omega _{n}$, of width $2\tau _{n}$ in research work of Gilles [26].

The empirical wavelet can be define as a bandpass filter for each $\Lambda _{n}$ by utilizing idea of both Littlewood-Paley amd Meyer’s wavelets [15]. The empirical scaling function can be defined as

1 $$\begin{aligned} \hat{\phi }_n(\omega )= {\left\{ \begin{array}{ll} 1 &{} \mathbf{if }\ |\omega |\le \omega _n-\tau _n \\ \cos \left[ \frac{\pi }{2}\beta \left( \frac{1}{2\tau _n}(|\omega |-\omega _n-\tau _n)\right) \right] &{} \mathbf{if }\ \omega _n-\tau _n \le |\omega |\le \omega _n+\tau _n \\ 0 &{} \mathbf{otherwise } \end{array}\right. } \end{aligned}$$

and the empirical wavelets can be given as follows:

2 $$\begin{aligned} \hat{\psi }_n(\omega )= {\left\{ \begin{array}{ll} 1 &\quad \mathbf{if }\ \omega _{n}+\tau _{n} \le |\omega |\le \omega _{n+1}-\tau _{n+1} \\ \cos \left[ \frac{\pi }{2}\beta \left( \frac{1}{2\tau _{n+1}}(|\omega |-\omega _{n+1}-\tau _{n+1})\right) \right] &\quad \mathbf{if }\ \omega _{n+1}-\tau _{n+1} \le |\omega |\le \omega _{n+1}+\tau _{n+1} \\ \sin \left[ \frac{\pi }{2}\beta \left( \frac{1}{2\tau _n}(|\omega |-\omega _n-\tau _n)\right) \right] &\quad \mathbf{if }\ \omega _n-\tau _n \le |\omega |\le \omega _n+\tau _n \\ 0 &\quad \mathbf{otherwise } \end{array}\right. } \end{aligned}.$$

The EWT of signal *f*(*t*), $W_{f}^{\varepsilon }(n,t)$, is defined the same as classic WT [26]. The detail coefficient is defined as

3 $$\begin{aligned} W_f^\varepsilon (n,t)=\langle f,\psi _n\rangle =\int f(\tau )\overline{\psi _n(\tau -t)}d\tau \end{aligned}$$

4 $$\begin{aligned} W_f^\varepsilon (n,t)=\langle f,\psi _n\rangle\,=\left( \widehat{f}(\omega )\overline{\psi _n(\omega )}\right) ^\vee \end{aligned},$$

where $\langle \rangle$ denotes inner product. Similarly, the approximation coefficient is defined as

5 $$\begin{aligned} W_f^\varepsilon (0,t)=\langle f,\phi _1\rangle =\int f(\tau )\overline{\phi _1(\tau -t)}d\tau \end{aligned}$$

6 $$\begin{aligned} W_f^\varepsilon (0,t)=\langle f,\phi _1\rangle\,=\left( \widehat{f}(\omega )\overline{\phi _1(\omega )}\right) ^\vee \end{aligned}.$$

The reconstruction of the signal *f*(*t*) can be obtained as

7 $$\begin{aligned} f(t)=W_f^\varepsilon (0,t)\star \phi _1(t)+\sum W_f^\varepsilon (n,t)\star \psi _n(t)\end{aligned}$$

8 $$\begin{aligned} f(t)\,=\left( \widehat{W_f^\varepsilon }(0,\omega )\phi _1(\omega )+\sum \widehat{W_f^\varepsilon }(n,\omega )\psi _n(\omega )\right) ^\vee \end{aligned}.$$

## Proposed feature extraction approach

Although EWT has been proposed by Gilles [26] for building adaptive wavelet to represent the signal to be processed, the author, however, has mentioned that the proposed method might fail to decompose properly when the input signal, like EEG signal (due to nature of multiple channels), compose of more than one chirp which overlaps in both time and frequency domain. As the performance of the classification model is highly dependent on the extracted features, features obtained using EWT from EEG signals are not suitable to produce an efficient classification model due to the problem mentioned above. Keeping this point into consideration, a very familiar fuzzy clustering method has been employed in this paper. The proposed method is able to deal with the problem of EWT by re-assigning the extracted features from EWT to the more similar type of segment using FCM algorithm. And this final processed signal will be able to produce good classification model. The brief description of FCM is given in the next subsection.

### Fuzzy C-means

Fuzzy C-means algorithm [27] is a clustering technique based on fuzzy set theory. Basically, fuzzy set theory is developed by Zadeh [28] and is viewed in different prospects by some researchers such as Nguyen [29] and Tiwari and Srivastava [30]. The core idea of FCM is that one object can belong in more than one cluster on the basis of fuzzy membership value ($\left[ 0,1\right]$) rather than on the ground of crisp value ($\lbrace 0,1\rbrace$) as in *k*-means algorithm. The non-linear optimization problem for FCM can be given as

9 $$\begin{aligned} {\left\{ \begin{array}{ll} \mathrm{Min}\ A_{m}(\mathbf {U},\mathbf {V};\mathbf {X}=\sum \nolimits _{j=1}^{p}\sum _{i=1}^{c}(u_{ij})^{m}d^{2}(x_{j},v_{i}))\\ \text {such that}\ \sum \nolimits _{i=1}^{c}u_{ij}=1,\ \ 1 \le j\le p \\ 0 \le u_{ij} \le 1,\ 1 \le j\le p,\ 1 \le i\le c \\ 0 \le \sum \nolimits _{j=1}^{c}u_{ij}<p, \ \forall i \end{array}\right. } \end{aligned},$$

where $\mathbf {X}=(x_{1},x_{2},...,x_{p})$ are *p* objects, $c\ (1<c<p)$ is number of the clusters, and $m\ (1<m<\infty )$ is fuzzifier constant. $u_{ij}$ is the degree value of membership of *j*th object to belong in *i*th cluster. $\mathbf {U}=(u_{ij})_{c\,\times \,p}$ and $\mathbf {V}$ are fuzzy partition and centroid matrix, respectively. Further, $d^{2}(x_{j},v_{i})$ denotes the Euclidean distance between *j*th object and *i*th centroid.

The updation of the fuzzy membership value of the given object after *k* iteration is given as

10 $$\begin{aligned} u_{ij}(k)=\frac{1}{\sum _{r=1}^c\left( \frac{d(x_j,v_i(k))}{d(x_j,v_r(k))}\right) ^\frac{2}{m-1}} \end{aligned}.$$

Similarly, the centroid point can be updated as

11 $$\begin{aligned} v_i(k+1)=\frac{\sum _{j=1}^pu_{ij}^{m}(k)x_j}{\sum _{j=1}^pu_{ij}^{m}(k)} \quad \mathrm{\ where}\ 1\le i\le c \end{aligned}.$$

### Feature coding

The proposed approach of extracting features from EEG signal is carried out in three steps. In the first step, the decomposition of the signal into desire number of support (segment) through the EWT is made. FCM clustering algorithm is employed in the second step of the proposed approach to avoid overlapping segments obtained from the first step. To represent each segment more compactly, eight statistical or uncertainty parameters (root mean square, Lempel–Ziv complexity measure [31], shannon entropy, central frequency, maximum frequency, variance, skewness, and kurtosis) have been calculated in the third or final step of the proposed technique as every signal or data have the distinguishable property in terms of a set of statistical parameters associated with the signal or data. It may be possible that the two signals have same value associated with one or more statistical parameter. In this work, these eight parameters are selected empirically.

## Feature selection

The feature vector from each channel obtained encloses all the features constructed with the above statistical parameters. The final feature vector obtained after concatenation of features from six channels is large, i.e., each feature vector contains 144 parameters (3 EWT segments $\times$ 8 parameters $\times$ 6 channels). Hence, feature selection is carried out to exclude noisy, irrelevant, and redundant features.

Two major categories of feature selection methods are the filter method and the wrapper method. In filter method, the relevance of features is determined on the basis of inherent properties such as distance, consistency, and correlation without involving any classifier. Hence, it may not choose the most relevant feature set for the learning algorithm. Alternatively, the wrapper method [32] has a tendency to find relevant features subset, better suited to a given learning algorithm. However, wrapper method is computationally more costly since the classifier needs to be learned for each feature subset separately. On the other hand, filter feature selection method is computationally less intensive and bias free. Filter methods have a simple structure with straightforward search strategy like forward selection, backward selection, or the combination of both.

Filter approach is further classified into two categories [20] as univariate (ranking) and multivariate (feature subset). A scoring function is used by feature ranking method for measuring the relevance of each feature individually. These methods are simple to compute. The research works have used univariate filter method in the BCI field [33–36]. It is noted that the reduced relevant features obtained from using univariate methods significantly improves the classification accuracy. But it ignores the correlation among the features. Hence, the selected feature subset may have high redundancy among features and may not provide high discriminatory capacity.

In the wrapper approach [37, 38], the seminal work of Keirn and Aunon [4] has used a combination of forward sequential feature selection and an exhaustive search to obtain a subset of relevant and non-redundant features for the mental task classification. However, wrapper approach is not suitable for high-dimensional data as it is computationally expensive.

On the other hand, efficient time multivariate filter method finds features which are relevant to the class and non-redundant among themselves. Thus, it overcomes the limitations of both univariate and wrapper approaches. Thus, we have preferred most widely used multivariate filter feature selection methods namely Bhattacharya distance measure [39], ratio of scatter matrices [40], and LR [41] for selecting relevant and non-redundant features. Brief discussion of these techniques is given below.

### Bhattacharyya distance

In the literature, BD is used as a dissimilarity measure between two probability distributions. It is a special case of Chernoff distance which measures the overlap between samples of two different probability distributions. For multivariate normal probability distribution, Chernoff distance measure is given as [42]

12 $$\begin{aligned} J_c=\frac{1}{2}\beta (1-\beta )(\varvec{\mu }_{2}-\varvec{\mu }_{1})^{T}[(1-\beta )\varvec{\Sigma }_{1}+\beta \varvec{\Sigma }_{2}]^{-1}(\varvec{\mu }_{2}-\varvec{\mu }_{1})+\frac{1}{2}\mathrm{log}\frac{\left| (1-\beta )\varvec{\Sigma }_{1}+\beta \mathbf {\Sigma }_{2} \right| }{\left| \mathbf {\Sigma }_{1} \right| ^{1-\beta }\left| \mathbf {\Sigma }_{2} \right| ^{\beta }} \end{aligned},$$

where ${\varvec{\mu }}_{i}$ and $\mathbf {\Sigma }_{i}$ are mean vector and covariance matrix for class $C_{i},$ respectively(*i*=1, 2).

When $\beta$ = $\frac{1}{2}$ then this distance is known as BD [39], which is given as

13 $$\begin{aligned} J_B=\frac{1}{8}(\varvec{\mu }_{2}-\varvec{\mu }_{1})^{T}\left( \frac{\varvec{\Sigma }_{1}+\varvec{\Sigma }_{2}}{2} \right) ^{-1}(\varvec{\mu }_{2}-\varvec{\mu }_{1})+\frac{1}{2}\log \frac{\left(\frac{\varvec{\Sigma }_{1}+\varvec{\Sigma }_{2}}{2}\right)}{\left| \varvec{\Sigma }_{1} \right| ^{\frac{1}{2}}\left| \varvec{\Sigma }_{2} \right| ^{\frac{1}{2}}} \end{aligned}.$$

However, it suffers from the problem of singularity when the determinant of covariance for a given class takes zero value.

### Ratio of scatter matrices

In the literature, a simple measure based on the scatteredness of features in high-dimensional space is recommended, which is a ratio of the trace of the SR. The measure selects those relevant features which are well clustered around their class mean and the means of two different classes of data are well separated. The SR, within-class scatter matrices, $\mathbf {S}_{w}$, and between class SR, $\mathbf {S}_{b}$, are defined as

14 $$\begin{aligned} \mathbf {S}_{w}=&\sum _{i=1}^{c}P_{i}E[(\mathbf {x}-\varvec{\mu }_{i})^{T}(\mathbf {x}-\varvec{\mu }_{i})]\end{aligned}$$

15 $$\begin{aligned} \mathbf {S}_{b}=&\sum _{i=1}^{c}P_{i}(\varvec{\mu }_{i}-\varvec{\mu }_{0})^T(\varvec{\mu }_{i}-\varvec{\mu }_{0}) \end{aligned},$$

where $\varvec{\mu }_{i}$, $P_{i},$ and $\varvec{\mu }_{0}$ are mean vector of *i*th class data, prior probability of $i^{th}$ class data, and global mean of data samples, respectively.

From the definitions of SR, the criterion value, which is to be maximized, is given as

16 $$\begin{aligned} J_{\mathrm{SR}}=\frac{trace(\mathbf {S}_{b})}{trace(\mathbf {S}_{w})} \end{aligned}.$$

$J_{\mathrm{SR}}$ takes high value when the inter-cluster distance is large and intra-cluster distance is small. The main advantage of this criterion is that it is independent of external parameters and assumptions of any probability density function. The measure $J_{\mathrm{SR}}$ also has the advantage of being invariant under linear transformation.

### Linear regression

Regression analysis is another well-established statistical method suggested in the literature that investigates the causal effect of independent variable upon dependent variable. The class label is used as the dependent variable (target), and the features that affect this objective are sought. The LR method attempts to find the linear relationship between a response variable and two or more explanatory variables by substituting a linear equation to the observed data. Since many features can affect the class, therefore multiple regression model is more appropriate. A multiple regression model with *k* independent variables $\mathbf {f}_1, \mathbf {f}_2, \ldots , \mathbf {f}_k$ and a target variable *y* is given by Park et al. [41]:

17 $$\begin{aligned} y_i=\beta _{0}+\beta _{1}f_{i1}+\cdots +\beta _{k}f_{ik}+\zeta _{i}, i=1,2,\ldots ,n \end{aligned},$$

where $\beta _{0},\beta _{1},\ldots ,\beta _{k}$ are constants estimated by class label *y* and observed values of $\mathbf {X}$. The sum of squared error (SSE) which is sum of the squared residuals is given by

18 $$\begin{aligned} \mathrm{SSE}=\sum _{i=1}^{n}(y_{i}-y_{i}^{p})^2 \end{aligned},$$

where $y_{i}$ and $y_{i}^{p}$ are target and predicated values, respectively. The smaller value of SSE shows better regression model. The total sum of squares (SSTO) is given by

19 $$\begin{aligned} \mathrm{SSTO}=\sum _{i=1}^{n}(y_{i}-\bar{y})^2 \end{aligned},$$

where $\bar{y}$ is the average value of $y_{i}, i=1,2,\ldots ,n$. The criterion value $J_{\mathrm{LR}}$ is given as

20 $$\begin{aligned} J_{\mathrm{LR}}=1-\frac{\mathrm{SSE}}{\mathrm{SSTO}} \end{aligned}.$$

The value of $J_{\mathrm{LR}}$ lies between 0 and 1. It considers a linear relationship between data and class labels. In a linear regression analysis, the feature for which the value of $J_{\mathrm{LR}}$ is higher is selected.

## Experimental setup and results

### Dataset

**Fig. 1:**
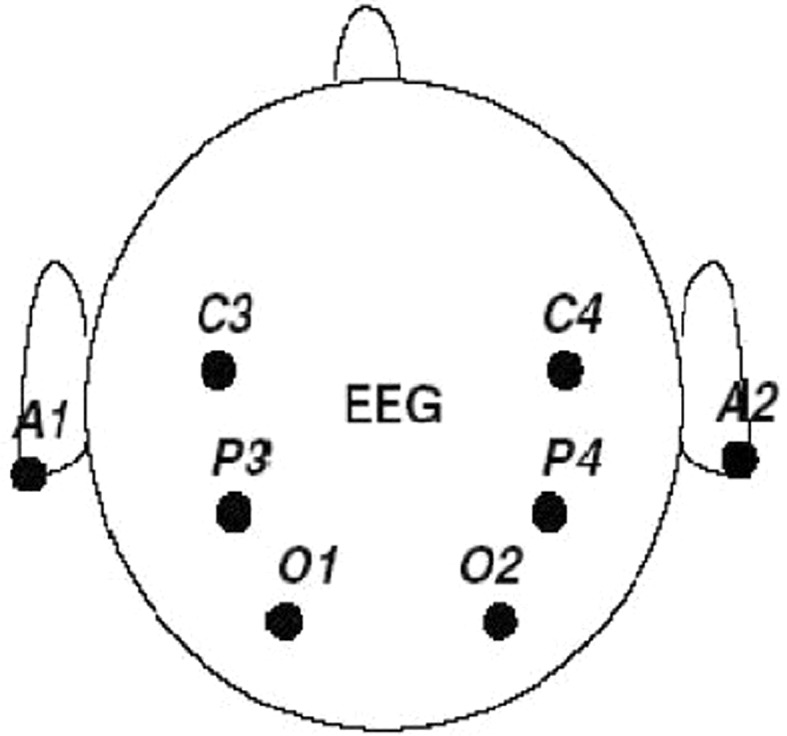
Electrode placement of EEG recording adapted from [13]

For our experiment, we have used publicly available data for mental task classification (Keirn and Aunon, 1990). The original EEG dataset consists of recordings from seven subjects, but we utilized data from all subjects except subject-4 due to some missing information. Each subject performed five different mental tasks: the baseline task (B)(no task); the mental letter composing task (L); the non-trivial mathematical task (M); the visualizing counting of numbers written on a blackboard task (C); and the geometric figure rotation task (R). Each of the recording session consists of five trials of each of the five mental tasks. EEG recording was taken from six electrodes placed on the scalp at C3, C4, P3, P4, O1, and O2 referencing to two electrodes placed at electrically linked mastoid, A1, and A2, as shown in Fig.  1.

Each trial is of 10 s duration recorded with a sampling frequency of 250 Hz, which resulted into 2500 samples points per trial. More detail about the data can be found in the work of Keirn and Aunon [4].^1^

### Construction of feature vector and classification

**Fig. 2:**
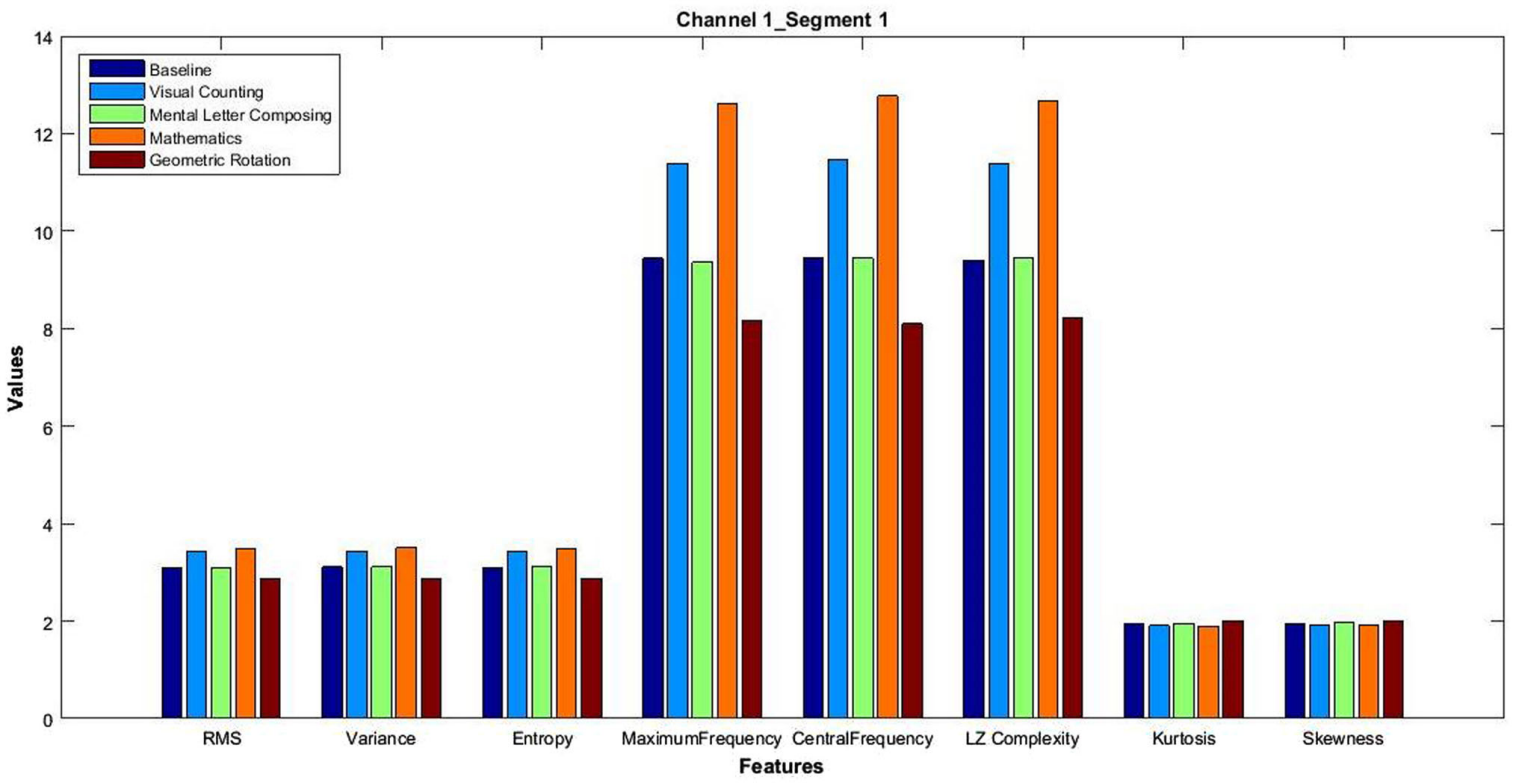
Eight features obtained for different tasks for channel 1 from segment 1 using FEWT for subject-1

For feature construction, the data are decomposed into half-second segments as some researchers have done [13], yielding 20 segments per trial for each subject. Features are extracted from each signal using three steps: in the first step signal is decomposed from three number of supports using EWT, in the second step, FCM clustering algorithm (with fuzzifier constant *m* = 2) is employed to form non-overlapping frequency bands and the final or third step the eight parameters are calculated. A total of 24 (3 $\times \,$8) features are obtained from each channel. Combining features of all six channels, each signal is represented in terms of 144 values. Further, it was observed during experiment that not all features were accountable for distinguishing two different mental tasks (see Fig. 2), and therefore, we have applied multivariate filter feature selection (BD, LR, and Ratio of SR) approach to select a set of relevant and non-redundant features.

For all the multivariate filter methods, the top 25 features were incrementally included one by one to develop the decision model of support vector classifier (SVC) using 10-fold cross-validation. We have used Gaussian Kernel. Grid search is used to find optimal choice of regularization constant *C* and gamma.

### Results

**Fig. 3:**
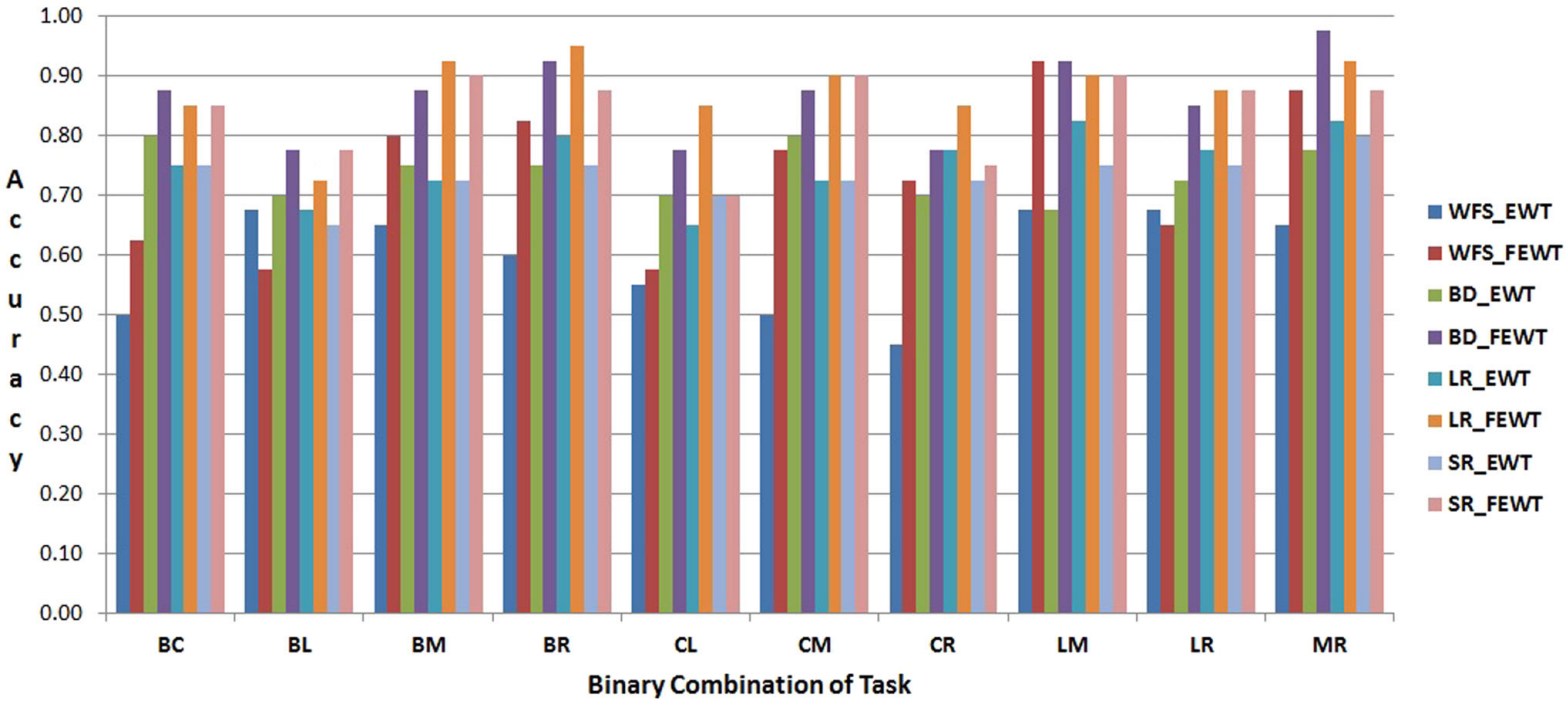
Performance of SVC for subject-1

**Fig. 4:**
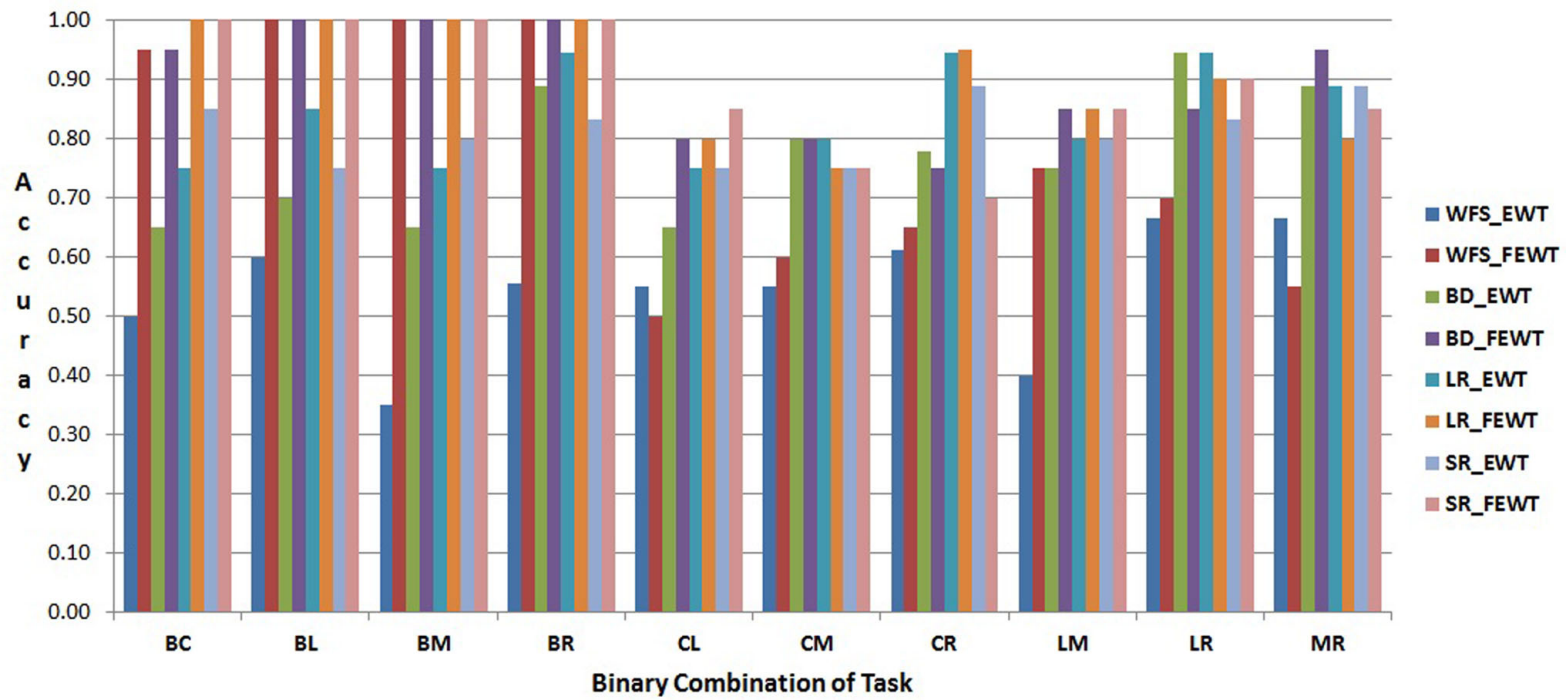
Performance of SVC for subject-2

**Fig. 5:**
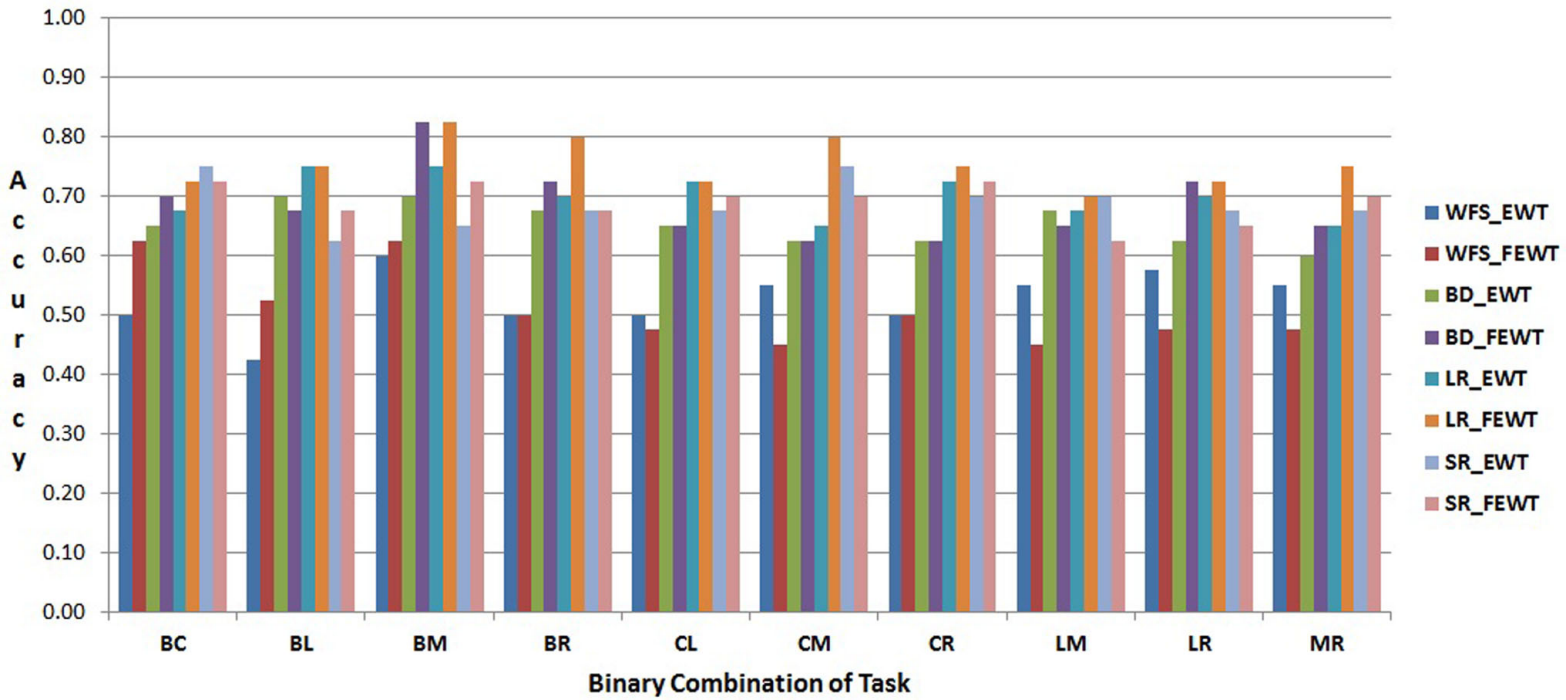
Performance of SVC for subject-3

**Fig. 6:**
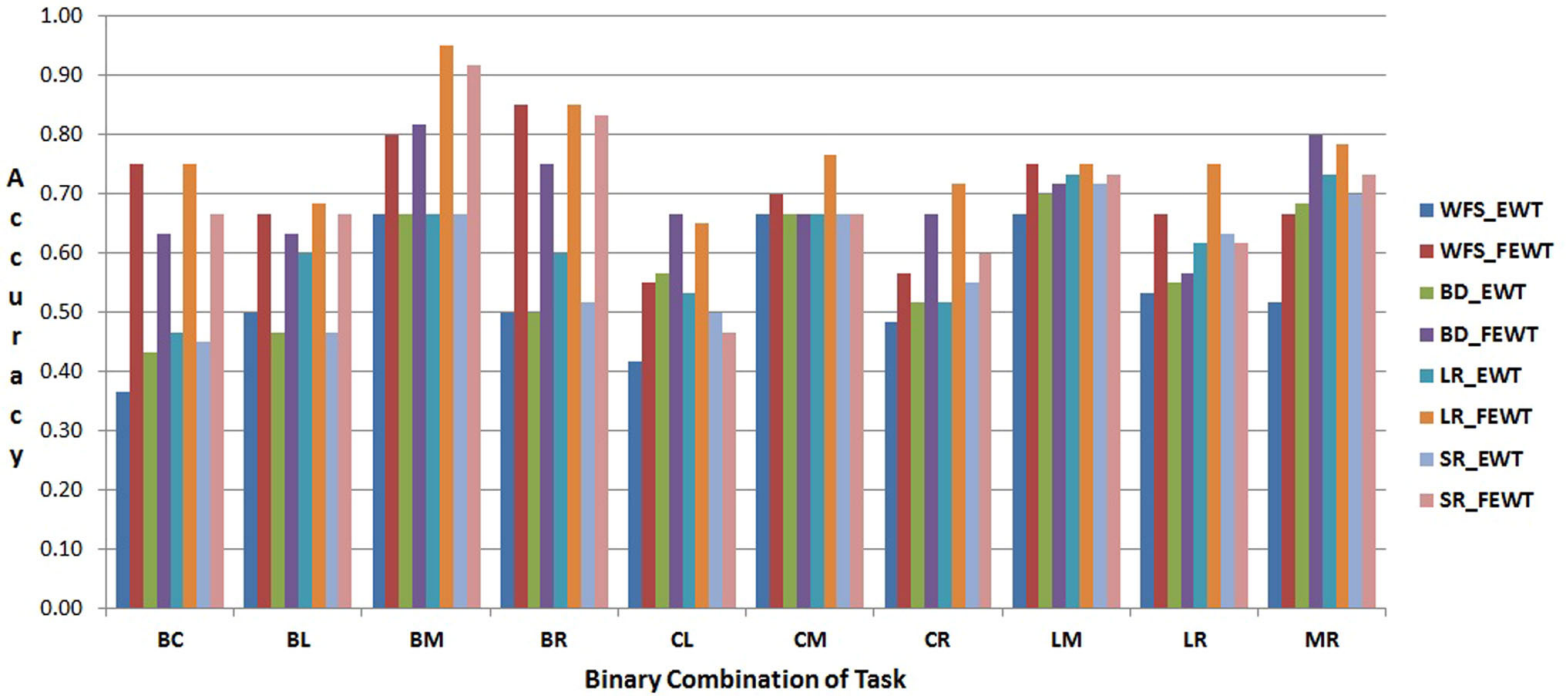
Performance of SVC for subject-5

**Fig. 7:**
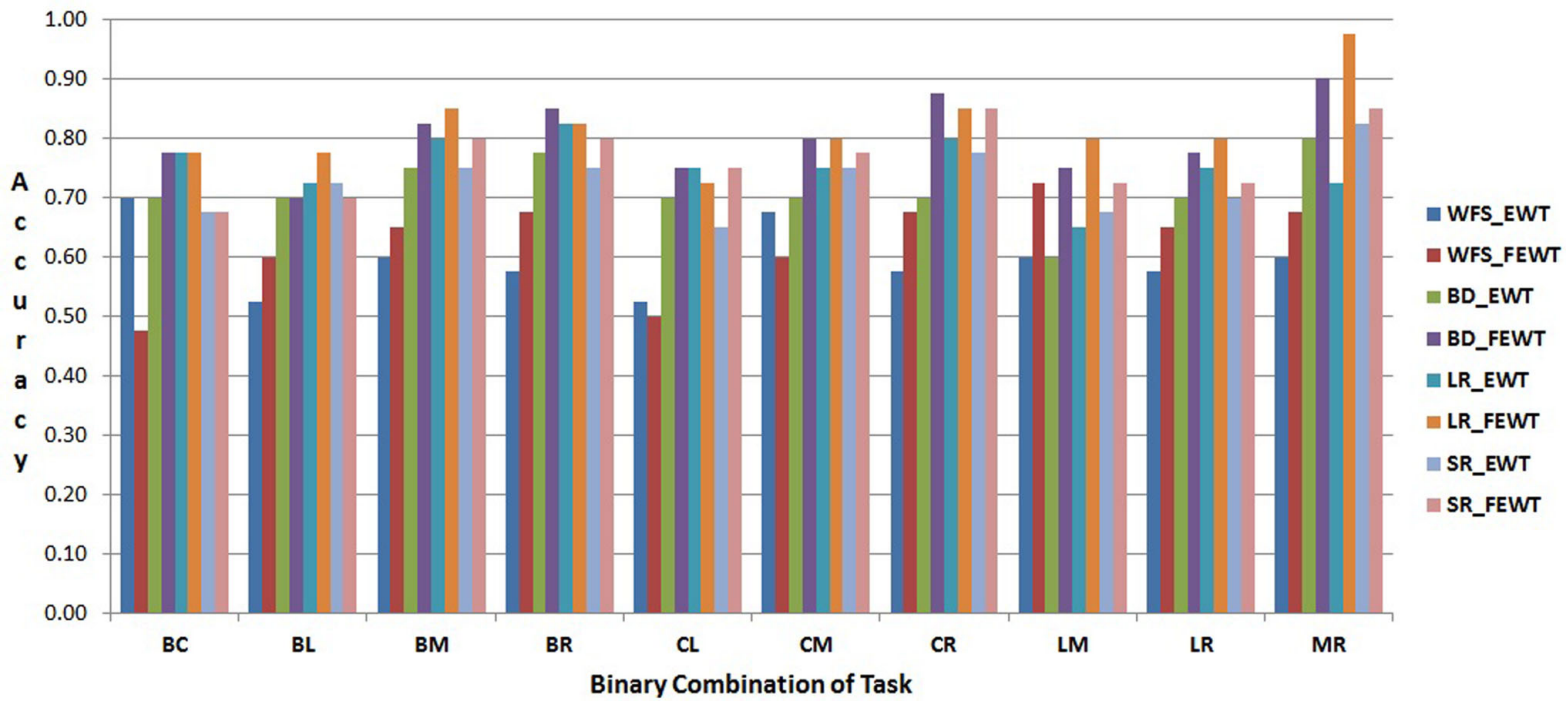
Performance of SVC for subject-6

**Fig. 8:**
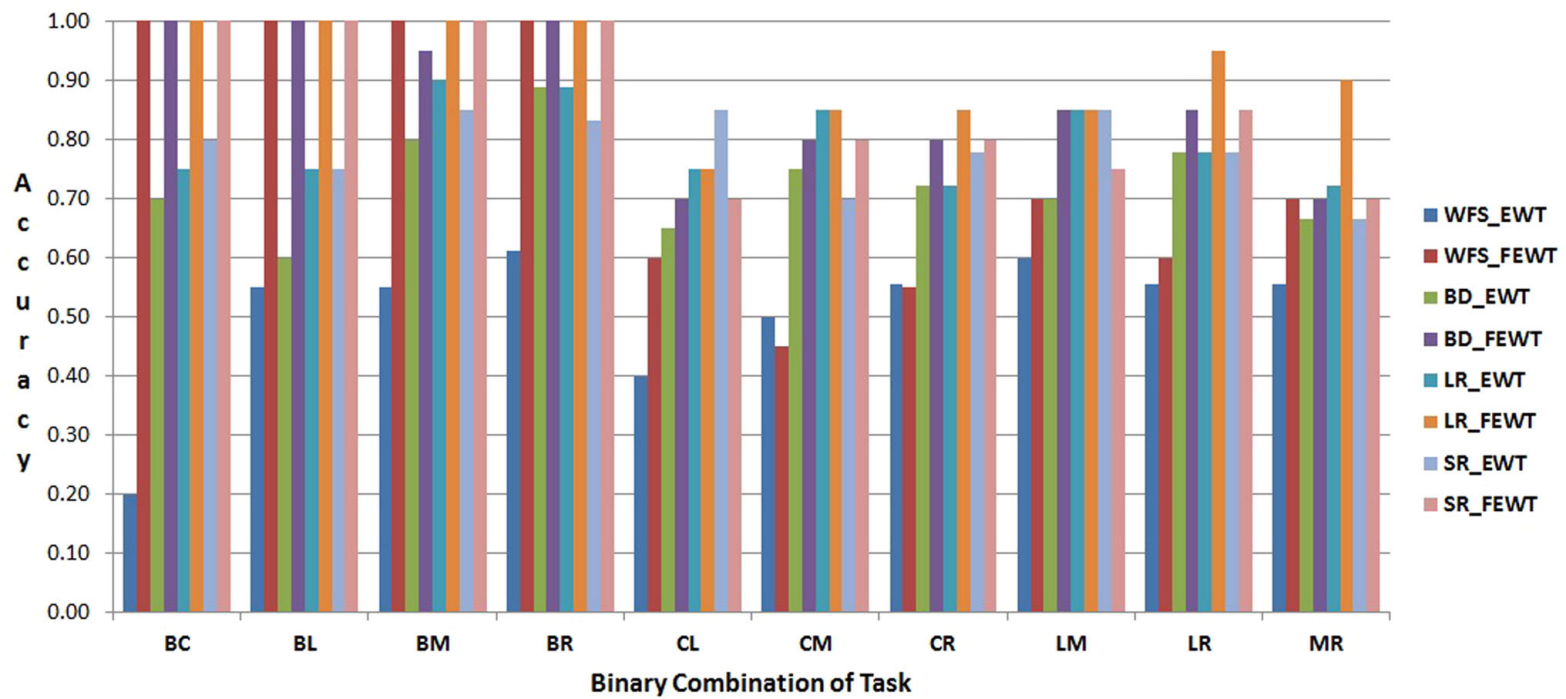
Performance of SVC for subject-7

The proposed FEWT methods is compared with the EWT method through the experimental setup as described above for binary mental task classification. Figures  3,  4,  5,  6,  7 and  8 show the classification accuracy taken overall average for the 10 binary combination of the five mental tasks for subject-1, subject-2, subject-3, subject-5, subject-6, and subject-7, respectively. From these figures, the following observations can be noted:

- The performance of classification model has significantly improved after incorporating the fuzzy clustering method along with the EWT compare to EWT alone irrespective of with or without feature selection method for all the binary combination mental tasks for all mentioned subjects.
- The classification accuracy of a given classifier has drastically increased with the application of feature selection methods (BD, LR, and SR) as compared to without feature selection (WFS) irrespective of feature extraction methods.
- From Figs.  4 and  8, for some binary combination of mental tasks 100 % classification accuracy for subject-2 and subject-7 is achieved.

### Ranking of various combinations of feature selection methods with proposed FEWT method

We have applied a robust ranking approach utilized by Gupta et al. [43], to study the relative performances of various combinations of feature selection methods with the proposed feature extraction method, i.e., FEWT with respect to EWT. To rank various combinations, the basis of percentage gain in classification accuracy with respect to maximum classification accuracy obtained using EWT feature extraction method with combination of various feature selection methods has been chosen.

A mathematical description of this ranking procedure is as follows:

If *i* = 0, then no feature selection is used; otherwise *i*th feature selection is used. $a_{\mathrm{FEWT}_{t}}^i$ denotes classification accuracy of *i*th feature selection method in combination with FEWT feature extraction method for *t*th task combination.

21 $$\begin{aligned}&a_{\mathrm{EWT}_{t}}^{\mathrm{max}}=\max \{a_{\mathrm{EWT}_{t}}^0, a_{\mathrm{EWT}_{t}}^1, a_{\mathrm{EWT}_{t}}^2,\dots , a_{\mathrm{EWT}_{t}}^i,\ldots, a_{\mathrm{EWT}_{t}}^n\}\end{aligned}$$

22 $$\begin{aligned}&P_{\mathrm{FEWT}_{t}}^i=\left( \frac{a_{\mathrm{FEWT}_{t}}^i-a_{\mathrm{EWT}_{t}}^{\mathrm{max}}}{ a_{\mathrm{EWT}_{t}}^{\mathrm{max}}}\right) 100 \end{aligned}.$$

Then the average (over all task combination) percentage gain in accuracy for *s*th technique is given by

23 $$\begin{aligned} P_{\mathrm{FEWT}}^{i}=\frac{1}{n_t}\sum _{t=1}^{n_t}P_{\mathrm{FEWT}_{t}}^i , \quad \forall i=0,1,2,...,n_s \end{aligned}.$$

Finally, the rank $r^s$ of each *i*th combination is assigned in such a way that

24 $$\begin{aligned} r^a\le r^b \quad if\ p^a\ge p^b \end{aligned}.$$

**Fig. 9:**
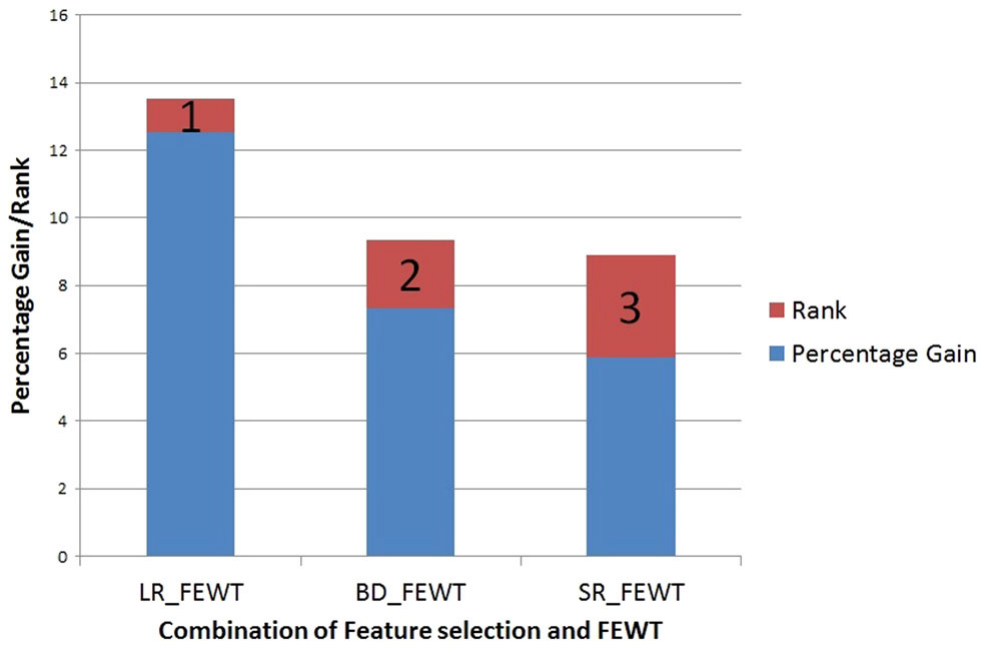
Ranking of combinations of feature selection methods with FEWT extraction method

Figure  9 shows four combinations of the feature selection and FEWT extraction methods compared against each other on the basis of percentage gain in accuracy. From Fig.  9, we can see the combination LR with FEWT acquires highest percentage classification accuracy gain with respect to the best combination of EWT with or without feature selection.

### Friedman statistical test

In order to determine the significant difference in various combinations of feature selection and EWT or FEWT statistically, we have applied a two-way [44] and non-parametric statistical test known as Friedman test [45]. Our null hypothesis $H_{0}$ was that there is no difference in performance among all combinations of feature extraction and feature selection. The alternative hypothesis $H_{1}$ was that there are differences among combinations. The $H_{0}$ was rejected at significant level *p* = 0.05. From Table  1, it can be noted that the combination of FEWT feature extraction and LR feature selection is the winner among all combinations of feature extraction and feature selection.

**Table 1:** Friedman ranking of different combinations of feature selection and extraction methods

Combination	Ranking
LR_FEWT	1
BD_FEWT	2.4
SR_FEWT	2.95
LR_EWT	4.3
WFS_FEWT	5.25
SR_EWT	5.75
BD_EWT	6.35
WFS_EWT	8

## Conclusion and future work

A theoretical adaptive transform, EWT, has been proposed in recent past to analyze signal on its content basis. EWT would fail to handle the signal which is overlapped in time and frequency domain as the case with the EEG signals from multiple channels. This work has suggested employment of FCM followed by EWT for better representation of EEG signal for further classification of mental task. It can be concluded from experimental results that the proposed approach outperforms as compared with the original EWT technique. It is also noted that the features from multiple channels generate a large size of the feature vector, but the available number of samples is small. Under such a situation, the performance of the learning model degrades in terms of classification accuracy and learning time. To overcome this limitation, this paper has investigated and compared three well-known multivariate filter methods to determine a minimal subset of relevant and non-redundant features. Experimental findings endorse that the employment of feature selection enhances the performance of learning model. Ranking mechanism and Friedman statistical test have also been performed for the strengthening the experimental findings.

As the employment of FCM enhances the performance of EWT technique for the mental task classification, it would be better to explore some other fuzzy-based clustering which has been explored in image segmentation [46]. It will also be interesting to explore whether the FEWT would work in other type of BCI such as motor imagery and multi-mental task classification.

## Footnotes

1
http://www.cs.colostate.edu/eeg/main/data/1989_Keirn_and_Aunon.
